# CRISPR/Cas9 and Nanotechnology Pertinence in Agricultural Crop Refinement

**DOI:** 10.3389/fpls.2022.843575

**Published:** 2022-04-08

**Authors:** Banavath Jayanna Naik, Ganesh Shimoga, Seong-Cheol Kim, Mekapogu Manjulatha, Chinreddy Subramanyam Reddy, Ramasubba Reddy Palem, Manu Kumar, Sang-Youn Kim, Soo-Hong Lee

**Affiliations:** ^1^Research Institute of Climate Change and Agriculture, National Institute of Horticultural and Herbal Science, Rural Development Administration (RDA), Jeju, South Korea; ^2^Interaction Laboratory, Future Convergence Engineering, Advanced Technology Research Center, Korea University of Technology and Education, Cheonan-si, South Korea; ^3^Floriculture Research Division, RDA, Wanju-gun, South Korea; ^4^CSSR and SRRM degree and PG College, Kadapa, India; ^5^Department of Medical Biotechnology, Dongguk University, Seoul, South Korea; ^6^Department of Life Science, College of Life Science and Biotechnology, Dongguk University, Seoul, South Korea

**Keywords:** Cas9, biotic and abiotic stress, horticultural crops, nutritional value, nanoparticles, nano-fertilizers, Cas9 activators

## Abstract

The CRISPR/Cas9 (Clustered Regularly Interspaced Short Palindromic Repeats/CRISPR-associated protein 9) method is a versatile technique that can be applied in crop refinement. Currently, the main reasons for declining agricultural yield are global warming, low rainfall, biotic and abiotic stresses, in addition to soil fertility issues caused by the use of harmful chemicals as fertilizers/additives. The declining yields can lead to inadequate supply of nutritional food as per global demand. Grains and horticultural crops including fruits, vegetables, and ornamental plants are crucial in sustaining human life. Genomic editing using CRISPR/Cas9 and nanotechnology has numerous advantages in crop development. Improving crop production using transgenic-free CRISPR/Cas9 technology and produced fertilizers, pesticides, and boosters for plants by adopting nanotechnology-based protocols can essentially overcome the universal food scarcity. This review briefly gives an overview on the potential applications of CRISPR/Cas9 and nanotechnology-based methods in developing the cultivation of major agricultural crops. In addition, the limitations and major challenges of genome editing in grains, vegetables, and fruits have been discussed in detail by emphasizing its applications in crop refinement strategy.

## Introduction

In agroecology, the integrated approach in production of crops and conceptual marketing management draws holistic economic concern. As reported by “Food and Agriculture Organization” (FAO, 2017), the crop failure be falls mainly due to biotic and abiotic factors, significantly influencing the economic values of crops (Gautam and Kumar, 2020). Environmental and climate changes, causing frequent flood, droughts, temperature variations, higher soil salinity, use of harmful chemicals as additives/fertilizers, pathogen triggered diseases, deteriorate the plant health and affects directly to the crop quality and yield (Bing, 2020). Typically, breeders follow customary methods along with marker-assisted selection to introduce new traits in plants. Some chemical compounds and irradiation techniques are also employed to attain desirable traits; however, they often lead to random mutations in crop genomes.

The customary methods have certain drawbacks such as non-specificity and the generation of mutations with abundance of nucleotides (Mao et al., 2019). Natural chemical compounds from plant source that are essential to the pharmaceutical industry can only be acquired in limited quantity from normal plants compared to genomically edited plants. Moreover, the rational methods are time-consuming; consequently, breeders are unable to grow plants with desired trait in deadline. Supplying sufficient food and other plant-based chemical constituents to ever-growing population is challenging; and quite perplexing topic in near future. The CRISPR/Cas9 genome editing (GE) with nanotechnology-based protocols can potentially challenge these obstacles.

The targeted genomic engineering can be extremely beneficial to agriculture. If the function of a specific gene is known, it can be over expressed or suppressed to obtain the desired trait. Eventually, the crops that are capable of withstanding biotic and abiotic stresses can be easily developed using CRISPR/Ca9 and nanotechnology. Similarly, undesired genes can also be silenced by using these technologies, which would allow expression of only the desirable traits to eventually obtain higher yield. It will be an extremely difficult task to improve crop refinement without genomic engineering. Conventional breeding programs would require longer duration to introduce new quality-related traits or disease-tolerance traits. Therefore, genomic engineering is a striking technology for the future development of agricultural crops with nutrition. Nanotechnology-based protocols has new sets of advanced applications in agriculture and biomedicine; typically, the nano-sized particles are used to deliver the task obtain desirable results in crop development. Coalescing biotechnology and nanotechnology approaches, including GE, have more benefits than customary breeding to improve the development of food crops, which can naturally overcome biotic and abiotic stress along with enhancing the yield. This brief review mainly focuses the importance of genomic engineering in agriculture, and the progress in developing mutant plants using sequence-specific nucleases (SSNs). Furthermore, the procreation of genomically edited crops are already developed in agricultural biotechnology, the main objective, limitations and prospective challenges of GE are highlighted including the role of nanotechnology-based methods in crop refinement.

## Generating Mutant Plants Using Sequence Specific Nucleases

Sequence specific nucleases are mainly used for precise gene editing in plants and animals. It can create mutations at desired loci in multiple genes *via* addition, deletion, and alternation of sequences (Songstad et al., 2017). To generate the mutant plants, firstly SSNs requires to be articulated in cells; subsequently, recognizing a specific DNA sequence to make the double stranded break. We can classify the SSNs into four major classes, namely (i) CRISPR-Cas9, (ii) Zinc finger nucleases (ZFN), (iii) Meganucleases, and (iv) Transcription activator-like effector nucleases (TALENs). These methods can be effectively utilized for the GE technique. CRISPR-Cas9 is derived from the adaptive immune systems of bacteria; in this mechanism, abounding components come into play to perform the GE. Zinc finger nucleases (ZFNs) are the enzymes that have been characterized 77 candidate two-finger modules (Urnov et al., 2005; Miller et al., 2007). Meganucleases are the microbial enzymes (Smith et al., 2006) that can 76 distinguish and more than 14 nucleotides for cleavage. TALENs have been developed by combining the *Fok*I nuclease domain with TALE proteins of *Xanthomonas* (Christian et al., 2010). CRISPR/Cas9 is one among the four classes of SSNs that can be used for GE (Songstad et al., 2017). GE technologies by adopting CRISPR/Cas9 methodology was investigated in 1987 and its functional application in human cell was reconnoitered in 2013 (Mali et al., 2013). Hsu et al. (2014) discussed the detailed challenges and its future prospective. Consequently, the GE technology was successfully implemented in crop refinement of soybean plant (Cai et al., 2015). Once the SSNs construct is incorporated into the plant genome, they are expressed at a distal site, while the remaining construct is removed by crossing the plant to obtain a mutated plant with no transgene. For GE with the CRISPR/Cas9 system, it is essential to deliver sgRNA and Cas9 proteins into the target cells. Expression vectors or microinjected RNA/mRNA (for Cas9) are usually used to express the sgRNA and Cas9 protein in the plant cells. The CRISPR/CAS9 technology has been performed in plant cells by using electroporation, *Agrobacterium*-mediated transformation, shotgun methodologies, and polyethylene glycol-mediated routes (Jiang et al., 2013; Li et al., 2013; Mao et al., 2013; Nekrasov et al., 2013; Shan et al., 2013). The double stranded break can be fixed either by homology directed repair (HDR) or non-homologous end joining (NHEJ) (Hsu et al., 2014). Recently, RNA viruses have been used to deliver hairpin RNAs for gene silencing, which is another reported technique to incorporate SSNs into the plant cells (Lacomme, 2015). Before the integration into plant’s genomic DNA, SSNs are transiently expressed from viral vectors into mRNAs and its respective proteins. The ability to modify the genes to modulate specific traits and homologous recombination (HR) allows plant to metabolize in a manner that develops their resistance to biotic and abiotic stresses. Plants synthesized by fast-growing genome engineering, generally exhibit higher crop yields because of its higher ability for photosynthesis. Precise altering the DNA sequence is extremely important to comprehend the achievable challenges insynthetic biology (Abdallah et al., 2014; Carroll, 2014).

### Components and CRISPR/Cas9 Mechanism

The guide RNA (gRNA) sequence comprising of twenty nucleotides that are essential to balance to the target DNA and the details of sgRNAs designing is well explained by Hussain et al. (2018). Similarly, the protein Cas9 has the catalytic activity and having the capability to cut the double standard DNA. When Cas9 and gRNA are combined to form a complex, cas9 immediately cuts the double-stranded DNA (Tang et al., 2019), and so forth the total gene sequence will be altered and specific protein synthesis will not befall by translation. There are two major pathways to repair the broken double-stranded DNA i.e., Non-homologous end joining (NHEJ) pathway and HDR (El-Mounadi et al., 2020).

### Non-homologs End Joining Pathway

The lost DNA part cannot be recollected in this NHEJ pathway. In this repair pathway, the dimeric protein complex (Ku) binds at the end of the broken DNA and later to DNA protein kinase catalytic subunits (DNA-PKCs). Artemis proteins are also come in to play and bind at the DNA terminal to make a complex; allowing phosphorylation and eventually the synthesis of DNA begin. This double-stranded DNA converted to blunt-ended double-stranded DNA by catalytic DNA ligase reaction, forming covalent linkage of phosphodiesters (Gomez et al., 2017). This repair system allows insertions or deletions of nucleotide bases that occur during a process (Bernheim et al., 2017; Tang et al., 2019).

### Homology Directed Repair Pathway

**FIGURE 1:**
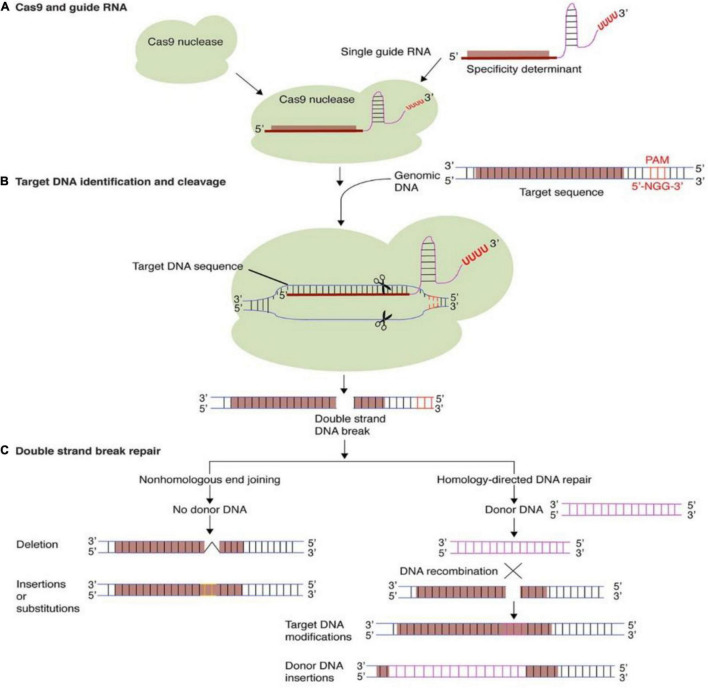
Targeted genome editing *via* CRISPR-Cas9. **(A)** The CRISPR-Cas9 system comprises of a Cas9 protein and guide RNA. Guide RNAs regulate the target DNA specificity by sequence complementarity. **(B)** gRNA and Cas9 protein form a binary complex that specifically cleaves target DNA creating a double-strand DNA break. **(C)** Cellular DNA repair mechanisms: non-homologous end joining (NHEJ) and homology-directed repair (HDR), repairs the double strand DNA break. In the process, short insertions, deletions, nucleotide substitutions, or gene insertion may occur. Reproduced with permission from El-Mounadi et al. (2020) Frontiers.

The HDR pathway use the autologous donor DNA sequences from sister chromatids or foreign DNA to create precise insertion and substitution between DNA double-strand break (DSB) sites for further alterations. Considerable research has been done previously on proteins involved in the HDR pathway, MRE11-Rad50-Nbs1 (MRN) complex binds at the 5′ end of the DSBs and forms the 3′ overhangs. Later the replication protein A (RPA) binds to the single-strand DNA to prevent the nuclease activity. RAD-51 protein involves in search of homologous DNA and eventually the invasion occurs to complete the homologs-directed repair (Tang et al., 2019). El-Mounadi et al. (2020) explained the CRISPR/Cas9 mechanism system was depicted in Figure 1.

## Importance of Genomic Engineering in Agriculture

Transgenesis profoundly involving genetic addition without changing the genetic pool to create specific traits for agricultural/agroeconomical benefits. In the present scenario, providing food security to a mounting populace is one of the major challenges in this modern world. Thenceforth, the food fructification needs to be increased over 80–90%. Furthermore, the food production is declining every year due to extreme weather conditions, climate change, global warming, farmland availability, and cumulative biotic and abiotic problems. To overcome these challenging obstacles, gene modification technique in food crops will be the superlative method to achieve the targeted tasks (FAO, 2017). The gene knockout step is a critical stage and it directly influence the phenotype. Wada et al. (2020) demonstrated genomic manipulation without the introduction of DSBs. In this modular approach, a dead Cas9 variant (dCas9) binds to the target sequence; however, it does not cleave the double-stranded DNA (Qi et al., 2013; Wang et al., 2016a; Adli, 2018). Generating gene knockouts using SSNs facilitates genetic analyses and the study of important gene functions, which will eventually help with crop improvement. The first mutation generated using SSNs were of the IPK1 gene in maize, which is catalyzed in the last step during phytate biosynthesis (Liang et al., 2014). Knocking out this gene helps in removing unwanted metabolites; eventually contributing to the accumulation of valuable biosynthetic intermediates. Prime editing is another significant approach for genomic manipulation that has been indicated in mammalian and yeast cells (Anzalone et al., 2019). Certain crops such as grains, vegetables, and fruits are vitally important to maintain global food securities and sustainable system.

Homologous recombination is a challenging process wherein chromosome can chasm by the nuclease that must be coordinated with the distribution of the DNA repair template. Initially, HR in plants was confirmed by the incorporation of marker genes at detailed chromosomal sites. Targeted transgene insertion into the euchromatin should provide promising results in plants with high transgene expression; therefore, it is a great improvement over the integration by the traditional Transgenesis (Neelendra et al., 2018). Moreover, the insertion of multiple genes at the same site will facilitate their transfer to a single mendelian locus, when the plant is crossed. Further efforts are required to familiarize plentiful genes into the germplasm by breeding. Targeted gene inclusion through HR using different SSNs has been verified in tobacco, maize, and rice (Abdallah et al., 2014; Wang et al., 2016b). In order to develop new plant varieties, some techniques have been used that are controlled by process-based regulatory frameworks. Most of the farmers, globally aim to cultivate crops that are tolerant to drought, high salinity, and diseases, with appreciable yield. Therefore, the researchers in agriculture domain are actively looking for transgenic technology and CRISPR/CAS9 to achieve targeted tasks. Guidelines and process-based regulations have been formulated and implemented by the United Nations Food and Agricultural Organization adopted by European and South American countries (FAO, 2017).

### Applications of Clustered Regularly Interspaced Short Palindromic Repeats to Develop Cereal Crops

Globally, there is a renowned demand for Basmati rice because of its fragrance, long grains, and flavor texture. However, due to bacterial blight caused by *Xanthomonas oryzae* pv. *oryzae* (Xoo), the yield and rice quality will be deprived from its authentic taste. By CRISPR/Cas9 GE technique, the genes namely OsSWEET11, OsSWEET13, and OsSWEET14 could be possibly edited to overcome the bacterial blight disease (Zafar et al., 2020). New Japonica rice is another prominent Asian rice variety, developed by editing Ehd1 (Early heading date 1) gene *via agrobacterium* mediated transformation. The mutated rice varieties exhibited prolonged basic vegetative growth (BVG) period at latitudes (Wu et al., 2020). It was documented that, *OsBADH2 (betaine aldehyde dehydrogenase*) *gene was modified to develop the* fragrance in non-aromatic rice *grains for better marketing* (Ashokkumar et al., 2020). Two endogenous genes, namely *TaWaxy* and *TaMTL*, were edited by using three different promotors (*OsU6a, TaU3*, and *TaU6*) for the development of haploid plants in wheat crops. Among these three promotors, *TaU3 showed better results* (Liu et al., 2020). TaPDS gene was edited in wheat by using Cas9 and Cpf1 (AsCpf1 and LbCpf1) nucleases in wheat (Kim et al., 2021). Lipoxygenases genes (GmLox1, GmLox2, and GmLox3) were edited by using CRISPR/Cas9 to afford lipoxygenase-free new mutant lines in soybean crop, so that it can be useful for human consumption with upsurge amount of protein for health benefits (Wang et al., 2020). Aside from maize, wheat, and rice, the fourth most highly consumable crop in the world is barley. The two mutated genes, HvHPT and HvHGGT in barley are mainly accountable for the dwindled grain size to weight ratios. Furthermore, these mutated barley lines showed reduced amount of tocotrienols assayed by HPLC (Zeng et al., 2020). Groundnut is the sixth most significant oil-seed crop in the world; this legume crop fixes the nitrogen *via* symbiotic relationship with rhizobia. In this process NFR (Nod Factor Receptor) play a major role in nitrogen fixation cycle. Thence, AhNFR1 and AhNFR5 genes were mutated by CRISPR/Cas9 to proliferate the root nodules *via* hairy root transformation system (Shu et al., 2020). CAD (cinnamyl alcohol dehydrogenase) and PDS (phytoene desaturase) genes were edited by CRISPR/Cas9 in sorghum *via* Biolistic bombardment resulted in enhanced biosynthesis of carotenoid and chlorophylls (Liu et al., 2019). SiMTL gene, which is orthologous to the maize *MATRILINEAL*/*NOT-LIKE-DAD*/*PHOSPHOLIPASE A* (*MTL*/*NLD*/*ZmPLA*) gene is edited by CRISPER/Cas9 with OsU3 promotor *via Agrobacterium* for the haploid induction in foxtail millet (Cheng et al., 2021). Please see Supplementary Table S1 (Fauser et al., 2014; Sugano et al., 2014; Baltes et al., 2015; Li et al., 2015, Li et al., 2016, 2018b,f; Li J. et al., 2017; Michno et al., 2015; Tsai and Xue, 2015; Xie et al., 2015; Zhou et al., 2015, 2016; Zhou X. et al., 2018; Baek et al., 2016; Duan et al., 2016; Feng et al., 2016; Gao et al., 2016, 2017; Iqbal et al., 2016; Osakabe et al., 2016; Pan L. et al., 2016; Pioneer, 2016; Pyott et al., 2016; Qi et al., 2016; Waltz, 2016; Watanabe et al., 2016; Zhang Y. et al., 2016; Zhao et al., 2016; Zhu et al., 2016; Chen et al., 2017; Kapusi et al., 2017; Mercx et al., 2017; Ordon et al., 2017; Wang et al., 2017b,c; Yang et al., 2017c; Che et al., 2018; Pankaj et al., 2018). For brief listing of prime cereal/food crop genes along with its specific functions modified *via* CRISPR/Cas9 system.

### Applications of Clustered Regularly Interspaced Short Palindromic Repeats to Develop Fruits/Vegetable Crops

Trans-Acting Small-interfering locus 4 (TAS4) and MYBA7 (Transcription factor) genes are edited by CRISPR/Cas9 *via* Agrobacterium to enhance the biotic and abiotic tolerance in grapes. These genes showed pronounced tolerance against the bacterium *Xylella fastidiosa* and *Grapevine Red Blotch* Virus (GRBV) causes Red Blotch Disease (Sunitha and Rock, 2020). Citrus canker is a dangerous disease caused by the bacterium *Xanthomonas axonopodis*. It is threatening to citrus family crops worldwide. The CsWRKY22 gene was edited by CRISPR/Cas9 with AtU6-1 promotor *via* Agrobacterium methodology to produce the Canker disease-free citrus in Wanjincheng orange plants. PDS (phytoene desaturase) gene was mutated for the development of Albino phenotype and carotenoid biosynthesis in banana crop (Otang Ntui et al., 2020). *Fusarium oxysporum* is a dangerous pathogen for watermelon. The editing of Clpsk1 gene that encode Phytosulfokine (PSK) precursor could be conferred to enhance resistance of *Fusarium oxysporum* to watermelon, the gene was efficiently edited by CRISPR/Cas9 system *via* Agrobacterium method to produce the *Fusarium oxysporum* resistant watermelons in appreciable yield (Zhang et al., 2020).

SlJAZ2 is a major co-receptor of coronatine (COR) in the stomatal guard cells of tomato fruit. This gene was edited for the development of bacterial speck disease resistance *via* Agrobacterium (Ortigosa et al., 2019). Broomrapes (*Phelipanche aegyptiaca* and *Orobanche* spp.), a kind of plant parasite can cause severe damage to the tomato plants. Thus, the CCD8 (Carotenoid Cleavage Dioxygenase 8) gene was edited to afford the *Phelipanche aegyptiaca* parasite resistant tomatoes (Bari et al., 2019). SlMlo1 and SlPelo genes were altered by CRISPR/Cas9 in tomato for resistant to yellow leaf curl virus and powdery mildew. These gene modifications successfully develop the pathogen-resistant tomatoes (Pramanik et al., 2021). Steroidal glycoalkaloids (SGAs) are plant secondary plant metabolites, better known for their toxic effects in humans and animals. High SGAs content can severely damage the potato quality. To diminish the SGAs content to optimum levels, StSSR2 (Sterol side chain reductase 2) gene, a key enzyme for the biosynthesis of SGAs was edited (Zheng et al., 2021). BoaCRTISO gene of Chinese kale was altered to increase the Carotenoid biosynthesis and the observed mutation rate was 81.25% (Sun B. et al., 2020). CsCRUC (*Camelina sativa* CRUCIFERIN C) gene encodes the seed proteins in *Camelina sativa* and this gene was edited by CRISPR/Cas9 to enhance the seed storage protein and enriched saturated fatty acids contents (Lyzenga et al., 2019). Powdery mildew is typically observed destructive disease that affect the leaves of wheat crop. This fungal leaf ailment can severely damage up to 40% of the crop under optimum ecological conditions (Gil-Humanes and Voytas, 2014). Thenceforth to overcome this problem MLO (MILDEW-RESISTANCE LOCUS) genes were modified in bread wheat (Wang et al., 2014). Assuredly, the bread wheat verities showed effective tolerance to powdery mildew. Bacterial blight disease is one of the most destructive afflictions, caused by *Xanthomonas oryzae* that can severely devastate for nutritional crop growth. In rice crops, the bacterial blight disease resistance was significantly improved by incorporating OsSWEET11, OsSWEET13, and OsSWEET14 genes (Zafar et al., 2020). To control the weed growth, the herbicide-resistant crops were instigated by OsPDS, OsPMS3, OsEPSPS genes (Zhang et al., 2014). The amylose content in rice endosperm was increased by the waxy (Wx) gene (Yunyan et al., 2019), thereby enhancing the grain number by OsSPL16 gene (Usman et al., 2021). Tiller-spreading phenotype of rice plants were improved by LAZY1 (LA1) gene (Miao et al., 2013). OsRR22 gene encodes 696-amino acid B-type response transcription factor that is intricate in cytokinin-signal transduction and metabolism, its loss of function suggestively upsurges the salt tolerance (Zhang A. et al., 2019) and the drought tolerance by OsNAC14 gene has been successfully developed CRISPR/Cas9 technique. For brief listing of prime fruits genes (see Table 1) and vegetable genes (see Supplementary Table S2; Ron et al., 2014; Butler et al., 2015; Ito et al., 2015; Lawrenson et al., 2015; Woo et al., 2015; Pan C. et al., 2016; Thomazella et al., 2016; Xu et al., 2016; Hayut et al., 2017; Hu et al., 2017; Klap et al., 2017; Koseoglou, 2017; Lang et al., 2017; Nonaka et al., 2017; Roldan et al., 2017; Soyk et al., 2017; Ueta et al., 2017; Yang et al., 2017b; Ye et al., 2017; Yu et al., 2017; Zhou et al., 2017) along with its specific functions modified *via* CRISPR/Cas9 system.

**TABLE 1:** Partial list of genes of fruit crops and its specific functions modified *via* CRISPR/Cas9 system.

S. No	Crop name	Gene name	Function	References
1	Grape	*VvPDS*	Carotenoid biosynthesis and albino phenotype	Nakajima et al., 2017
2		*IdnDH (L-I donate dehydrogenase)*	Promotes tartaric acid accumulation	Ren et al., 2016
3		*VvWRKY52*	Resistance to necrotrophic fungal pathogen Botrytis cinerea	Wang et al., 2018a
4		*ALS1*	Motifs characteristic of a cell surface protein to enhance the adherence to epithelial cells	Osakabe et al., 2018
5		*CsLOB1*Promoter	Enhanced resistance to citrus canker	Jia et al., 2016
6		*TAS4b* and *MYBA*	Biotic and abiotic stress tolerance	Sunitha and Rock, 2020.
7		*MLO-7*	Increased resistance to fire blight disease	Malnoy et al., 2016
8	Citrus	*PDS and Cs2g12470*	Albino phenotype	Jia et al., 2017; Zhu et al., 2019
9		*CsLOB1*	Resistance to canker disease	Peng et al., 2017; Jia et al., 2019
10		*CsWRKY22*	Resistance to canker disease	Wang et al., 2019b
11		*DMR6*	Huanglongbing (HLB) tolerant	Zhang X. et al., 2018
12	Sweet Orange	*CsPDS*	Carotenoid Biosynthesis	Jia and Wang, 2014
13	Apple	*ALS1*	Encodes cell surface protein to enhance the adherence to epithelial cells	Osakabe et al., 2018
14		*DIPM-1, 2*, and *4*	Increased resistance to fire blight disease	Malnoy et al., 2016
15	Apple and Pear	*MdPDS* and *Md TFL1*	Early flowering phenotype	Charrier et al., 2019
16	Straw berry	*AP3 (APETALA3)*	Control of flower development	Martín Pizarro et al., 2019
17		*FvARF8 and FveTAA*	Auxin biosynthesis	Zhou J. et al., 2018
18		*PDS*	Albino phenotype	Wilson et al., 2019
19	Kiwi	*AcCEN4* and *AcCEN*	Rapid terminal flower and fruit development	Varkonyi-Gasic et al., 2018
20		*PDS*	Carotenoid biosynthesis and albino phenotype	Wang et al., 2018b
21	Banana	eBSV	Resistance to banana streak virus	Tripathi et al., 2019
22		*PDS*	Carotenoid biosynthesis and albino phenotype	Kaur et al., 2018; Otang Ntui et al., 2020
23		*MaGA20ox2*	Regulates semi-dwarf	Shao et al., 2019
24	Watermelon	*ALS*	Conferring herbicide resistance	Tian et al., 2018
25		*ClPDS*	Carotenoid biosynthesis	Tian et al., 2017
26		*ClPSK1*	Resistance to *Fusarium oxysporum*	Zhang et al., 2020
27	Papaya	*alEPIC8*	Resistance to *Phytophthora palmivora*	Gumtow et al., 2018

### Applications of Clustered Regularly Interspaced Short Palindromic Repeats to Develop Ornamental Crops

Ornamental plants usually grown for decoration aspirations and are often associated with commercial orientations in agroecological farming practices. These attractive flowering plants are customarily utilized in extracting perfumes and opens a profitable platform in fragrance market. Ornamental plants also a play a vital economic role in farming economy and sustainable agricultural business. Marigold flower and its extracts are used in poultry industry as feed additive, in order to enhance the quality of egg production and enticing yolk color. PhNR (Petunia Nitrate Reductase) gene was modified to check the nitrogen uptake and nitrate metabolism in petunia plants. Mutated plants showed better nitrogen uptake efficiency (Subburaj et al., 2016). *CiPDS* (chicory *phytoene desaturase*) gene was edited by under U6 promotor *via* Agrobacterium mediated and protoplast transfection methods. Among these two methods, Agrobacterium mediated route showed 31.25% transformation efficiency in chicory and the mutated chicory plants showed better Albino phenotype content (Bernard et al., 2019). MADS genes (MADS, MADS44, MADS36, and MADS8) from flowering plant of the orchid genus Phalaenopsis was using CRISPR/Cas9 for floral initiation and flower development (Tong et al., 2020). *Lilium* spp. is a genus of more than 100 species of flowering plants emergent from bulbs. To intensify the beauty and color shades, LpPDS gene was edited by CRISPR/Cas9 (Yan et al., 2019), to enforce to bloom attractive pale yellow to albino–green color shades. Table 2 gives the concise listing of prime genes of fruits and its specific functions modified *via* CRISPR/Cas9 system.

**TABLE 2:** Partial list of genes of ornamental plants and its specific functions modified *via* CRISPR/Cas9 system.

S. No	Crop name	Gene name	Function	Reference
1	Petunia	*PhPDS (Phytoene Desaturase)*	Albino phenotype	Zhang B. et al., 2016
2		*PhNR (Nitrate reductase)*	Nitrogen uptake and nitrate metabolism	Subburaj et al., 2016
3		*PhACO1, 3*, and *4*	Flower longevity and the reduction in ethylene production	Xu et al., 2020
4		*PiSSK1*	S-RNase-based self-incompatibility mechanism	Sun and Kao, 2018
5	Japanese morning glory	*InDFR-B*	Floral color change	Watanabe et al., 2017
6		*InCCD4 (Carotenoid Cleavage Dioxygenase)*	Carotenoid accumulation and floral color change	Watanabe et al., 2018
7		*EPH1*	Delays petal senescence	Shibuya et al., 2018
8	Chicory	*CiPDS*	Display an albino phenotype	Bernard et al., 2019
9	*Chrysanthemum morifolium*	*CpYGFP(Yellowish-green Fluorescent)*	Disruption of fluorescence protein	Kaboshi et al., 2017
10	*Lilium pumilum*	*LpPDS*	Display an albino phenotype	Yan et al., 2019
11	*Phalaenopsis equestris*	*MADS, MADS44, MADS36*, and *MADS8*	Flower initiation and development	Tong et al., 2020
12	Wishbone Flower	*F3H (Flavanone 3-hydroxylase)*	Flavonoid biosynthesis and initiating catalysis of the 3-hydroxylation of (2S)-flavanones	Nishihara et al., 2018
13	*Camelina sativa*	*FAD2*	Enhancement of fatty acids, especially linoleic acid	Jiang et al., 2017
14	*Lotus japonicus*	*SYMRK, LjLb1, LjLb2*, and *LjLb3*	Efficient inactivation of symbiotic nitrogen fixation	Wang et al., 2016c
15	*Dendrobium officinale*	*C3H, CCR, 4CL, C4H, and IRX*	Reduced lignocellulose biosynthesis	Kui et al., 2017
16	Easter lily (*Lilium longiflorum*)	*LlPDS*	Pale yellow and albino–green chimeric mutants	Yan et al., 2019
17	*T. fournieri*	*TfRAD1*	Diverse pigmentation patterns and petal shape regulations	Su et al., 2017
18	Red sage	*SmCPS1*	Tanshinone biosynthesis	Li B. et al., 2017

## Important Considerations for CRISPR/Cas9 Genome Editing

Genomic engineering has been encountered broad range of applications to introduce targeted alterations of the plant’s genome to acquire desired function (Hsu et al., 2014). The NmeCas9 (*Neisseria meningitidis*) recognizes an 8-mer PAM (5′-NNNNGATT) sequence hence it can progress the target particularity, whereas SaCas9 recognizes a 6-mer PAM sequence (5′-NNGRRT) (Xing et al., 2014; Ma et al., 2015). This technique involves introducing mutations, and harnessing transgene supplementation for gene therapy and livestock improvement. CMV (Califlower mosaic Virus), AtUBO (Arabidopsis UBO) OsUBO (Oriza sativa UBO), LTR (Long terminal Repeat), OsnoRNA U3 (*Oriza sativa* snoRNA U3), AtU6 (Arabidopsis U6), OsUBI (*Oriza sativa* Ubiquitin), ZmUBI (*Zea mays* UBI), cauliflower mosaic virus 35S promoters have been used to promote Cas9 expression in plants and, more than 30 empty gRNA backbones in binary vectors was supplied by Addgene (Belhaj et al., 2013; Feng et al., 2013; Mao et al., 2013; Nekrasov et al., 2013; Upadhyay et al., 2013; Xie and Yang, 2013). Tissue specific promoters can also be used in CRISPR/Cas9 technology to edit genomes; for instance, Wang et al. (2015) used the promoter of the egg cell-specific EC1.2 gene, to initiative of Cas9. The germ line-specific SPOROCYTELESS and embryo-specific promotor DD45 (Mao et al., 2016). The promotor AtDMC1 involved in meiotic recombination (Xu et al., 2018). The pLAT52-GT for Pollen tissues (Mao et al., 2016), and pDD45-GT for egg cell-early embryo tissues (Mao et al., 2016), INCURVATA2 for dividing tissue-targeted site-directed mutagenesis (Hyun et al., 2015), and the YAO promoter for cell-division specific tissues (Yan et al., 2015). In order to knock out the expressions of multiple genes cassettes can be inserted into one plasmid, thereby guiding the Cas9 to different targets (Xing et al., 2014; Ma et al., 2015; Oz et al., 2021). Although this GE technology has key advantages, there are some negative shades involving ethical issues concerning to the disruption of ecological balance (Tavakoli et al., 2021). To overcome this, different methods including nanotechnology-based methods are being implemented to insert or silence the genes in plant cells (Nadakuduti and Enciso-Rodríguez, 2021).

## Emphasis of CRISPR-Cas9 in Nutrition and Healthcare

Despite animal-based food consumption, the global ecosystem mainly be contingent to the agro-based crops including herbivorous animals. In concern to this, extensive research findings are mainly focusing on developing nutritional cereal/vegetables/fruits/nuts (FAO, 2017). Cultivating nutritional crops mainly depends on the nature and the fertility of the soil. Sources of soil nutrients are not same and is mainly depending on the presence of organic matter. In order to overcome this nutrient deficiency, CRISPR/Cas9 technology is extremely useful to grow cereal/vegetables/fruits/nuts with high nutritional values. Recently, genomic engineering utilizes TALENs, Cas9, dCas9, and Cre inserting into the cells. The use of these proteins in human cell lines have also been verified *in vitro* and *in vivo* (Zuris et al., 2015). HR (Sun et al., 2016) mediates the modifications made by the engineered nucleases. To date, plentiful of food-based crops have been modified to obtain good nutritional values in vegetables and fruits (Kathleen, 2015). Varieties of fruits comprised of different nutrients and biologically active compounds that are necessary in daily healthy diet. Recently, Dalla et al. (2019), Wan et al. (2021), and Xu et al. (2021) have discussed the importance of gene editing in fruits and vegetables to get nutritional rich cereal/vegetables/fruits/nuts, which are beneficial to maintain good health.

Genome-wide association studies (GWAS) have been employed to identify specific locations in the genome that can anchorage polygenic diseases such as Alzheimer’s, diabetes, autism, and schizophrenia. This technique is crucial in biomedical field to treat various diseases, including the removal of HIV genome (Ebina et al., 2013; Liao et al., 2015; Kaminski et al., 2017). The *ex vivo* and *in vivo* GE of neurons, immune cells, and endothelial cells has been successfully reported in mice (Platt et al., 2014), which are challenging to modify the sensitive cells and its effective editing. Researchers have introduced resistance against malaria by editing the DNA in *Anopheles mosquitoes* (Gantz et al., 2015; Hammond et al., 2016; Macias et al., 2020). Cancer therapy strategies have also been conducted in biomedical field to treat cancerous cell lines (Yao et al., 2015; Khan et al., 2016; Yang H. et al., 2019). Furthermore, AIDS research is still ongoing to engraft Cas9-modified CCR5-human hematopoietic stem cells and progenitor cells (Mandal et al., 2014). GE techniques are gaining its impact in promising therapeutics in regenerative medicine. The primary route for disease treatment is direct GE in tissues; some reports have documents the correction of monogenic recessive genetic disorders, such as Duchenne muscular dystrophy (Ousterout et al., 2015), hemophilia (Park et al., 2016) cystic fibrosis (Schwank et al., 2013), and sickle cell anemia (Sun and Zhao, 2014). The Cas9 system has exhibited its efficacy in therapies through the insertion of SSNs into microbial populations using phages and conjugative plasmids (Citorik et al., 2014).

## Developments and Possibilities for Genomic Engineering in Agriculture

The comprehensive study on rice (Shan et al., 2013), sorghum (Jiang et al., 2013), tobacco (Li et al., 2013), wheat (Wang et al., 2014), tomato (Brooks et al., 2014), maize (Liang et al., 2014), sweet orange (Jia and Wang, 2014), and Arabidopsis (Li et al., 2013), stretches the vast knowledge of specific gene handling and editing. Before SSNs, RNAi technology was used to study the gene function by knocking down the targeted genes, which was not as advantageous as SSNs. Certain characteristics discussed below encompasses the examples for certain horticultural and ornamental plants using the CRISPR system. The color and weight/size ratio of tomato fruit could be developed by the editing of PL and TBG4 genes (Wang et al., 2019a). The SlNPR1 and SlCBF1 genes corresponding to drought and cold tolerance can also be modified (Li et al., 2018b; Li R. et al., 2019) and the fruit ripening transcription factor RIN (Ripening Inhibitor) could be edited, so that the tomato with desired characteristics will be maintained. Albino phenotype and flowering characters could be modified in cabbage using FRI and PDS gene editing (Murovec et al., 2018). In addition, the biosynthesis of Carotenoid pigment can be enhanced in wild cabbage with BoaCRTISO (Carotenoid isomerase) gene editing (Sun B. et al., 2020). By editing DcF3H and DcPDS, DcMYB113 genes, the accumulation of acylated anthocyanins can be enriched in the roots of carrot to afford pigmented purple carrots (Chodacka et al., 2018; Xu et al., 2019). Cucumber mosaic virus (CMV-Z1) and Zucchini yellow mosaic virus (ZYMV) are two major rapidly affecting pathogens, which can severely damage the crop. To overcome this, the pathogenic resistance can be developed/enhanced by editing elF4EF gene (Chandrasekaran et al., 2016). Drought tolerance in an important commercial crop, hot pepper (*Capsicum annuum* L. syn. chilli) was developed by editing NAC72 gene (Joshi, 2019). Furthermore, to enhance the flower longevity of attractive petunia flowers can be edited using PhACO1, 3, and 4 gene (Xu et al., 2020). The color and the Carotenoid accumulation of Japanese morning glory flower can be edited by altering its related gene InCCD4 (Carotenoid Cleavage Dioxygenase) (Watanabe et al., 2018). Compatibly, the flavonoid biosynthesis could be enriched in Wishbone flower by editing F3H (Flavanone 3- hydroxylase) gene (Nishihara et al., 2018). Moreover, the AhFAD2A and AhFAD2B genes encoding fatty acid desaturases in groundnut have been reported to be edited (Yuan et al., 2019). The TYLCV-IR (Intergenic regions) gene has been modified to overcome the multiple viral diseases in *Nicotiana benthamiana.* After gene modification plant exhibited resistance to *geminiviridaevirus*, *begomovirus*, *curtovirus*, *becurtovirus*, *eragrovirus*, *Turncurtovirus*, and *Topocuvirus* (Ali et al., 2015a,b). The resistance against *Phytophthora tropicalis* in cacao has been overcome by altering the TcNPR3 gene (Fister et al., 2018). Giovannini et al., 2021 have identified the desirable phenotypic characters in ornamental flowers, including flowering induction, floral meristem initiation and organ development, as well as color, fragrance, and shelf life. Flower longevity has been induced in petunia by altering a group of Petunia hybrid 1-aminocyclopropane-1-carboxylate oxidase (PhACO, PhACO1, PhACO3, and PhACO4) genes (Xu et al., 2020). Canker and huanglongbing diseases are the major factors in reducing the productivity of citrus plants; this problem was overcome by modifying the CsLOB1, CsWRKY22, and DMR6 genes by CRISPR/Cas9 system (Peng et al., 2017; Zhang et al., 2018c; Wang et al., 2019b). Selectable marker gene (SMG) systems are critical and play a major role in the identification of transgenic crops. Nowadays, the scientists are considering the SMGs that can affect human and animal health. The gene transferred plants (GMP: Genetically Modified Plants) usually contain the antibiotic resistant gene, so that GM plants should survive and regenerate under antibiotic medium. Whereas, the non-gene transformed plants will not rejuvenate, eventually die under toxic proximity. Although antibiotics have positive health and life prospective in human/animal health, the negative impacts of antibiotic associated diarrhea and pseudomembranous colitis will proliferate the possibilities of subsequent diseases. By consuming those GMP (Fruits and vegetables) for prolonged usage can severely affect the human/animal health (Breyer et al., 2014). Thus, it is imperative to eliminate SMGs from transgenic crops by using CRISPR technology (Yau and Stewart, 2013). Arndell et al. (2019) edited the biosynthesis of 5-enolpyruvylshikimate-3-phosphate synthase (EPSPS) for the functional confirmation of EPSPS gene in wheat using CRISPR/Cas9. The soybean storage protein genes were also been edited successfully to observe the efficacy of CRISPR/Cas9 technique using Agrobacterium rhizogenes-mediated hairy root transformation method (Li C. et al., 2019). Acetolactate synthase (ALS) participates in amino acid biosynthesis; this amino acid is targeted by numerous herbicides. These two plant enzymes(EPSPS, ALS, ACCase), and BFP genes, confer herbicide tolerance in plants (Voytas and Gao, 2014; Sauer et al., 2016; Li et al., 2018e). Hybrid paddy are susceptible to bentazon and sulfonylureas and the BEL gene has been mutated using radiation. In the production of hybrid rice, these mutants can be used to prevent contamination in hybrid seed lots (Cantos et al., 2014). Here, the BEL gene was edited using CRISPR-Cas9 technology, and transformed into rice through *A. tumefaciens* (Xu et al., 2014). Nutrient values have also been increased in vegetables and fruits by knocking out genes using the CRISPR-Cas9 system. Visually attractive flowers possess pleasant aroma because of the presence of anthocyanin, whose expression is regulated by the MYB-bHLH-WD (MBW) complex (Albert et al., 2014; Lloyd et al., 2017). Gibberellin (GA) determines plant height and strigolactone (SL) affects branching of the shoot branching, both of which can be modulated by modifying the biosynthesis or signal transduction of GA and/or SL (D’Halluin et al., 2008).

Unwanted metabolites usually have negative impacts on the crop yield and its quality; the accumulation of these undesired metabolites can be avoided by using GE. Cyanide intoxication, ataxia or partial paralysis, and goiters are caused by cyanide, which is present in cassava (Padmaja, 1995). Glucosinolates, which produced by mustard and cabbage, also possess a high toxic content, were edited (Hannoufa et al., 2014). FAD2 and FAD3 genes produce high oleic acid and low linolenic acid in soybean; however, soybean oil allows the accumulation of monounsaturated fats and reduces the linolenic acid in the seeds (Pham et al., 2012). AtPDS3, AtFLS2, AtADH, AtFT, AtSPL4, and AtBRI1 genes are targeted in Arabidopsis with mutation rates (MRs) from 1.1 to as high as 84.8% in the first generation (Li et al., 2013). The OsPDS and OsBADH2 genes have been knocked out with MRs of 9.4 and 7.1% (Hinge et al., 2015). The DsRED2, DD20, and DD43 genes have been targeted in sorghum with MRs of 33, 59, and 76%, respectively. Similarly, the ZmIPK (13.1%), LIG1, MS26, MS45, and ALS1 genes have been edited in maize with MRs lower than 5% (Liang et al., 2014; Svitashev et al., 2015). TaMLO-A, TaMLO-B, and TaMLO-Dare three homeo alleles that confer powdery mildew resistance and have been edited with the same moderate MR of 5.6% (Wang et al., 2014). In BRI1, JAZ1, and GAI genes mutation frequencies of 26–84% have been observed (Feng et al., 2013). NtPDS and NtPDR6 have been mutated with MRs of 81.8 and 87.5%, respectively (Gao et al., 2015). The squamosa promoter binding protein-like 4 and Flowering Locus T (FT) have been mutated with an MR of 90%, which caused it to exhibit late flowering (Hyun et al., 2015).

**FIGURE 2:**
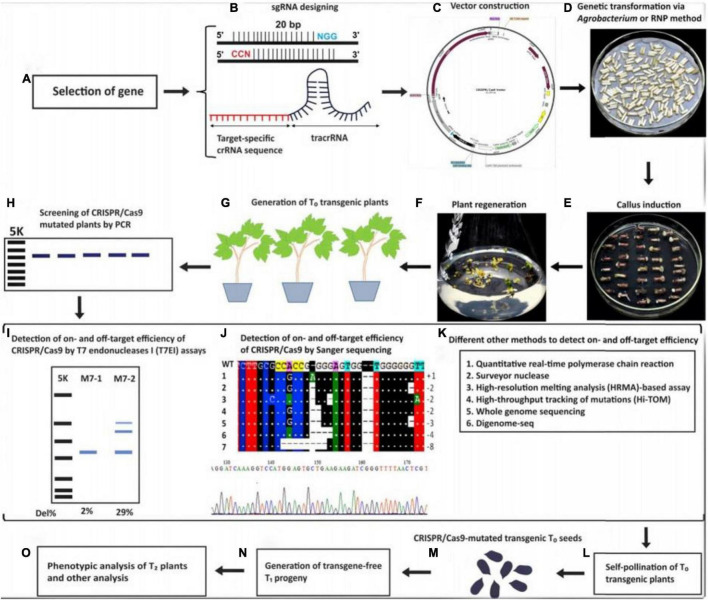
Schematic illustration of the steps involved in CRISPR/Cas9 Genetic Transformation; **(A)** Specific gene selection, **(B)** sgRNA designing for the specific gene, **(C)** vector construction, **(D)** transformation of the CRISPR/Cas9 system *via Agrobacterium*, **(E)** callus induction from agrobacterium infected explants, **(F)** plant regeneration from callus, **(G)** T_0_ CRISPR/Cas9 mutated transgenic plants, **(H)** screening of transgenic plants by PCR, **(I)** identification of mutated plants by T7E1, **(J)** detection of transgenics by sanger sequencing, **(K)** various techniques to detect transgenic plants, **(L)** self-pollination of T_0_ transgenic plants for generation of homozygous T1 plants, **(M)** mutated T_0_ seeds, **(N)** Generation of transgene- free T_1_ progeny, **(O)** Phenotypic analysis of T_1_ plants. Reproduced with permission from Manghwar et al. (2019) CellPress.

Ma et al. (2015), have reported genome modifications at 46 target sites with an average of 85.4% mutations in monocot and dicot plants using either golden gate ligation or Gibson assembly. Using the sgRNA single, double and triple mutants have also been generated for CDKA2, CDKB1, and CDKB2 in rice (Endo et al., 2015). Dort et al. (2020) discussed forest pathosystems; some disease problems were solved using the CRISPR/Cas9 system. Zhang et al. (2014), have also reported mutations in young seedling albino (OsYSA) and OsROC5 genes with MRs of 65–66.7%. Similarly, Wang et al. (2016b), have edited the OsERF922 gene that encodes ERF transcription factors to develop resistance to rice blast disease. Transgenic poplar plants have been modified and phenotypically results revealed an MR of 51% (Fan et al., 2015). El-Mounadi et al. (2020) explained biosafety of genomically edited plants and the applications of CRISPR/CAS9 technology to enhance yield, quality, and nutritional values. The crops and seeds developed using CRISPR/Cas9 technology for cereals, vegetables, ornamental, and fruits plants are shown in Tables 1, 2 and Supplementary Tables S1, S2. Some multinational companies (DuPont, Monsanto, and BASF) had obtained licenses to develop new crops using CRISPR technology (Khurana and Rajarshi kumar, 2019). The sequential steps for CRISPR/Cas9 genetic transformation in plants was sketched in Figure 2 (Manghwar et al., 2019).

## Role of Nanotechnology in Crop Refinement

**FIGURE 3:**
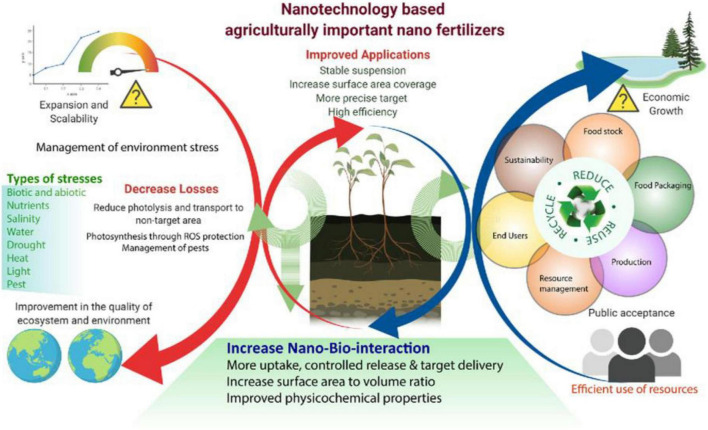
Nanotechnology-based agriculturally important nano-fertilizers, which are increasing the agronomic productivity, efficiency, and reduce environmental stress. Although showed the improved applications in agriculture by nanotechnology and Types of stresses overcome by nanotechnology. Reproduced with permission from Mittal et al. (2020) Frontiers.

Nanotechnology plays a prominent role in biological, medicinal, and pharmaceuticals including plant science. Increasing resilience to biotic and abiotic stress and improving the yield/quality of the crops *via* gene editing, nanotechnology shares its connotation with CRISPR/Cas9. Nano-fertilizers (Subramanian et al., 2015; Thul and Saragani, 2015) are used in horticultural plants, including vegetables and fruits, and implemented in food crops to enhance the growth, germination rate, and genetic manipulations (Lee et al., 2010; Sheykhbaglou et al., 2010; Dimkpa et al., 2012; Shang et al., 2019; Mittal et al., 2020; Aqsa et al., 2021; Rana et al., 2021). Similarly, plant growth, nutrient uptake from roots, flowering; have also been developed by metal and carbon based nanoparticles (Aqsa et al., 2021). Photosynthesis is an energy conversion process in plants, transforming light energy into chemical energy; however, it does not occur effectively under cloudy conditions and in sun-drenched plants during the rainy season. Consequently, cell mechanisms possibly down regulated. The gold nanoparticles could be beneficial to enhance the light-harvesting capacity, thereby promoting highly excited electron transfer in the chloroplast (So et al., 2015). Environmental factors (abiotic stress) cause biochemical and physiological changes in plants and these are more susceptible to stress. Even in stressful conditions, the use of metallic nanoparticles can increase the anti-oxidative enzyme levels in plants (Mittal et al., 2020; Zhao et al., 2020; Wu and Li, 2021) and reduce the reactive oxygen species (ROS) levels in the mitochondria and chloroplasts to protect the plant (Sun L. et al., 2020; Sun et al., 2021). However, the use of these nanoparticle fertilizers in crop fields not only increase the soil fertility, but also greatly influence the water resource contamination (Naderi and Abedi, 2012; Mittal et al., 2020). Fertilizers containing microorganisms are labeled as biofertilizers, which can activate the plant system and improve the nutrient uptake from soil (Manikandan and Subramanian, 2016). Nano-fertilizers have the similar benefits like biofertilizers (Elias et al., 2019). Moreover, metallic nanoparticles have anti-pathogenic, antifungal, and antibacterial properties (Kah and Hofmann, 2014; Servin et al., 2015), so that they can survive under pathogenic bout under the soil. Brief explanation of using metallic nanoparticles in farming and its benefit in sustainable agriculture is explained in Figure 3 (Mittal et al., 2020).

## Role of Nanoparticle-Based Genetic Modification of Crops

**FIGURE 4:**
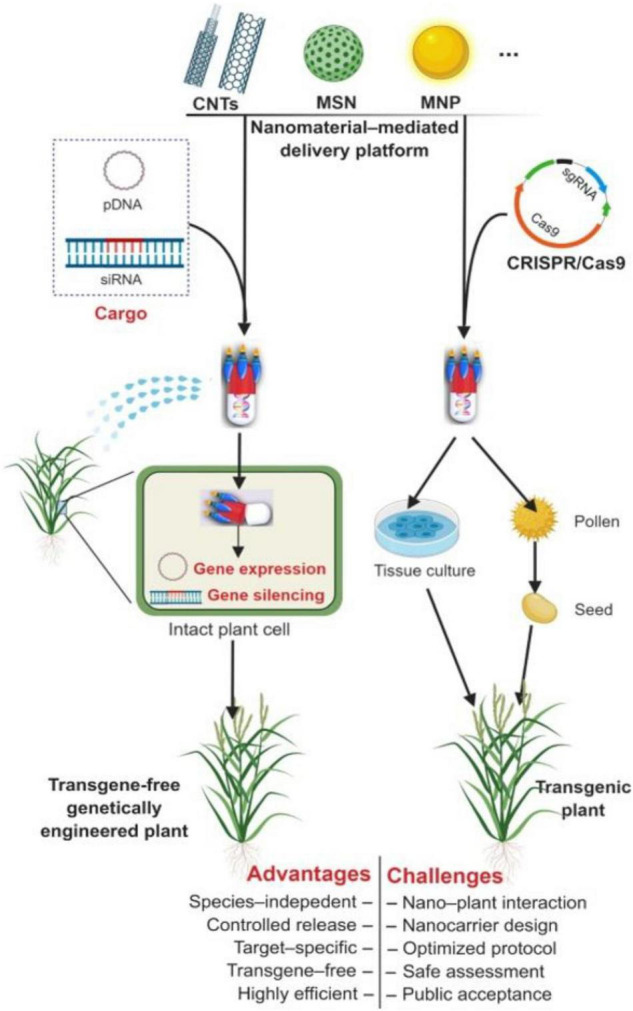
Nanomaterial-mediated plant genetic engineering. Functionalized nanomaterials can provide a delivery platform that is capable of traversing barriers (e.g., multilayered cell walls) to deliver exogenous plasmid DNA (pDNA) and siRNA into intact plant cells. CNTs, carbon nanotubes; MSN, mesoporous silica nanoparticles; MNP, magnet nanoparticles. Reproduced with permission from Peng et al. (2019) CellPress.

Nanoparticles acts as a carrier to deliver the necessary materials into plant cells, animal cells, and specific organs for cancer therapy, genetic disease treatment, and to obtain desirable traits in plants (Peng et al., 2019). Lv et al. (2020) and Ahmar et al. (2021) explained the method in which the gene transformation is performed using nanoparticles. Various types of nanoparticles have been used to deliver genetic material into the plant cells through different platforms. These reports have also detailed the merits and demerits of utilizing nanoparticles in gene transfer methods. Typically, the mesoporous silica nanoparticles, carbon nanotubes, gold, and magnetic nanoparticles have been used to deliver plasmid DNA, double-stranded RNA, and siRNA into plant protoplasts or other intact cell lines (Al-Babili and Bouwmeester, 2015; Yao et al., 2016; Peng et al., 2019; Huan et al., 2020; Tsveta et al., 2021). However, Lv et al. (2020) have demonstrated the gene silencing and gene editing in plants with the use of nanoparticles; magnetic nanoparticle-based pollen transformation was used to achieve the task. In this approach, the vector-magnetic nanoparticle complex has been associated with the pollen that is dropped onto the stigma of desired plant flowers. Finally, plants produce the desired seeds by transferring the vector-magnetic nanoparticle complex into the flower stigma. Later, these flowers are modified into fruits, and the seeds were screened on antibiotic plates. The speed breeding protocol was used to obtain T0, T1, and T2 generations of transgenic plants; this breeding program is inexpensive for editing plant genomes and is employed for various *Brassica* species (Ahmar et al., 2021). Similarly, dsRNA has been loaded into the layered double hydroxide (LDH) clay nanosheets that are non-degradable, non-toxic, and resistant to easy wash. Furthermore, when these nanoparticle-dsRNA complexes are sprayed onto the plant leaves, they immediately attach to the leaf surface and are absorbed by plant viruses to induce RNAi, eventually degrading the targeted plant pathogens or endogenous mRNA can be minimized/eliminated (Lv et al., 2020). Similarly, gene editing has also been demonstrated with small NPs-CRISPR/Cas9 vector complex that was microinjected into the leaves or any other plant parts, which can be proliferated further by tissue culture or other ease protocols (Lv et al., 2020; Duan et al., 2021). Carbon dots-siRNA complex has been used to silence the GFP in tobacco and tomato plants (Schwartz et al., 2020). Demirer et al. (2021) recently demonstrated genome editing in plants using the CRISPR/Cas9 system along with nanoparticles and explained the regeneration, and phenotypic/metabolic changes of genomically edited crops. The methods in which gene expression, silencing, editing, and other applications involving nanoparticles can account for the crop refinement and are briefly explained in the Figure 4 (Peng et al., 2019).

## Applications and Limitations of CRISPR/Cas9 and Nanotechnology Systems in Modern Biology

The CRISPR/Cas9 and nanotechnology aspects were successfully implemented so that relevant solicitations in plant biotechnology, food industry, livestock improvement can be achieved (Islam et al., 2020). Biofuel production (Javed et al., 2019), and rectifications of genetic and metabolic diseases can be done by controlling bacterial and viral diseases (Robert et al., 2017; Zafar et al., 2020), controlling bacterial and viral diseases (Zafar et al., 2020).

(1) This GE technology has been used to treat hematologic diseases, infectious diseases, and malignant tumors *via* gene therapy.
(2) Gene pyramids and gene knockouts can be created by inserting foreign genes through this approach.
(3) Repression/activation of gene expression.
(4) Delivery of proteins to genomic loci.
(5) Gene expression can be regulated with this technology. It also plays a major role in the progress of new mutant diversities to help the breeders.
(6) Gene silencing is an important aspect in the crop improvement program based on CRISPR intrusion (CRISPRi) (Larson et al., 2013; Qi et al., 2013).
(7) It is a promising technique to study the new gene’s function.
(8) Plants that are lenient to biotic and abiotic stresses, as well as herbicides, can be easily developed.
(9) Researchers are already using this technology in gene therapy (Qian et al., 2019; Li et al., 2020).
(10) Currently, nanotechnology is being used to prepare nano-fertilizers, nano-pesticides, and to enhancing the abiotic stress tolerance, thereby protecting the crops.
(11) Different types of NPs have also been used for the transformation, editing, and silencing of genes to improve crop yield and quality.

CRISPR/Cas9 technology is among the best and most cost-effective methods for genomic editing in plants. However, some of its limitations are mentioned below.

(1) Genomic editing is inefficient and slow by the presence of larger protein sizes. Therefore, protein size should be small to ensure speedy and efficient genomic editing.
(2) It contains limited number of PAM sites at limited loci.
(3) CRISPR/CAS9 can introduce multiple random mutations, as well as mutations at unspecific loci.
(4) It has low HDR efficiency.
(5) It exhibits low efficiency against viruses.
(6) It has become difficult to commercialize transgenic crops developed using CRISPR/Cas9 technology in many countries because different countries have different rules and regulations.
(7) In backward and developing countries, there is a lack of proper understanding of pesticides, fertilizers, and other products developed by nanotechnology; therefore, it is impossible to grow better crops.

### Challenges

At present, this technology is helping researchers in agriculture and breeders in developing crops that can overcome biotic and abiotic stress, with high nutritional values, and optimal yield parameters, thereby providing adequate food grains, vegetables, and fruits to current world population. Even with the availability of all types of biotechnological and bioinformatics tools, there are enduring challenges in developing genetically modified plants using the CRISPR system. Owing to unavailability of complete genome sequence information and the large genome size of some tropical, medicinally valuable crops, and fruits, some studies have been unable to edit genes to obtain desirable traits (Sanskriti et al., 2019; Zhou et al., 2019; Yang, 2020). Therefore, the biological pathways of genes and their interactions with environmental factors are still unknown (Haque et al., 2018). In order to discover new traits in tropical and other crops, one must be well-versed in the functions and regulatory elements of each gene. Moreover, in some crops, the transformation efficiency is extremely low; therefore, it takes a long time for the regeneration of explants, which is very difficult in some crops (Altpeter et al., 2016; Sanskriti et al., 2019). However, genetically modified crops require extensive field trials (Syed et al., 2020). Of all the challenges facing in the development of genetically modified plants, lack of proper public acceptance has become the biggest challenge (Yang, 2020), and believing to be accepted in forthcoming decades (Chao et al., 2021).

## Conclusion and Future Perspective

Scientists and plant breeding researchers are working to develop high yielding and biotic/abiotic stress resistant variety. CRISPR/Cas9 GE in consolidation with nanotechnology have emerged as an important platform to improve the quality and desirable quality of agricultural crops with appreciable yield. These technologies are significantly susceptible and open new prospects in plant genetics. The nutritional values and quality of health prospects of plants and human could be enriched with broad spectrum of applications including in biomedical domain. The imminent fertility and diminishing useful prokaryotic microorganism issues in soil are delicate issues in addition to biotic problems. Relapsing the aforesaid issues could be feasible using CRISPR/Cas9 GE and nanotechnology aspects, it will be challenging and greatly influence to the crop refinement. Skillful editing of genetic sequence of plant genome could progressively improve the agronomic traits, photosynthetic capacity, and nutritional values. Furthermore, biotic and abiotic stress-induced issues in plants could be configured and minimized. The methodologies can also be diversified to characterize the individual gene functions, thereby improving the genomic sequences of agricultural crops to produce exceptional yield. These findings could point new strategies in agroecology facilitating the sustainable production of nutritional quality food to satisfy the increasing demand of ever-growing population.

CRISPR/Cas9 and nanoparticle complex system is an advanced innovative technique in agricultural crop development/refinement. Thus far, CRISPR system in connotation with nanotechnology has been used to improve the quality and yield of many valuable crops for future benefits. Using nanoparticle based fertilizers/additives; extinct nutrients could be regained, ensuring that the soil nutrients could be maintained. The green, non-toxic nanoparticles is a sagacious approach to increase micro and macronutrient levels in the soil for healthy growth of crops. Chemically derived nanoparticle usage should be eliminated; these can harm not only the crops but also to the environment. Zinc oxide-based nanocomposites and nano-fertilizers have shown appreciable results in crop growth; maintaining the soil salinity and fertility with robust yield. The green protocols to develop Zinc oxide-based green nanocomposites draws the attention of agro-scientific community and it should be given prime importance, so that toxic free effective nano-priming techniques can reduce the soil contamination and improves the seed quality. Metal organic frameworks (MOF) have been recently investigated as delivery systems for CRISPR/Cas9. Non-toxic and eco-friendly MOF 3-dimentional structures with biopolymer conjugates are potentially promising. Furthermore, CRISPR/Cas9-associated nanoparticle complex has been successfully utilized for transformation, silencing, and modification of genes to overcome the existing and expected critical biotic and abiotic issues, thereby producing nutritional-rich crops. This technology can be used to alter the metabolic pathways in plants to obtain desired high-quality secondary metabolites for future usage. Recently, revolutionary changes in this crop refinement program witnessed auspicious results. Nod factors have been shown to increase nitrogen efficiency in legume crops using CRISPR/Cas9 system with nano-technological contrivance. Modifying the Nod signaling pathway in cereal crops should eliminates the use of toxic inorganic fertilizers. The transformation efficiency by *Agrobacterium tumefaciens* is quite low in some specified plant tissues. Hence, an alternative bacterial system to gene transfer is necessary for ease genome editing in all crops by adopting CRISPR/Cas9-Nanotechnology system. Furthermore, the development of tissue culture free delivery protocols involving direct genomic editing in germplasms and meristematic cells of plants, can yield propitious results. To date, numerous crops have been developed with this combined CRISPR/Cas9-Nanoparticle complex system. It is advisable to framework on the products already developed by this method and ensure their recurrent use in agricultural locales. Extensive studies are recommended to elucidate the complete interactions (plant cell mechanisms) of nanoparticles/nanocomposites in all types of crops. We strongly believe that the products developed by conjoining these technologies will beneficially assist the agro-based researchers to bloom their ideas for innovative crop development/refinement.

## Author Contributions

BN planned the manuscript outline, wrote the draft, and prepared the tables. GS contributed in writing, reshaping/editing the manuscript and modifying the tables. GS, S-CK, MM, CS, RP, and MK proofread the manuscript. S-YK acquired the funding. S-YK and S-HL supervised the study and revised the manuscript. All the authors reviewed and approved the final version of the manuscript.

## Conflict of Interest

The authors declare that the research was conducted in the absence of any commercial or financial relationships that could be construed as a potential conflict of interest.

## Publisher’s Note

All claims expressed in this article are solely those of the authors and do not necessarily represent those of their affiliated organizations, or those of the publisher, the editors and the reviewers. Any product that may be evaluated in this article, or claim that may be made by its manufacturer, is not guaranteed or endorsed by the publisher.
