# Supplementary material for: CRISPR/Cas9 and Nanotechnology Pertinence in Agricultural Crop Refinement
Source: Front Plant Sci. 2022 Apr 8;13:843575. doi: 10.3389/fpls.2022.843575 (PMC9024397; doi:10.3389/fpls.2022.843575)
Supplement: Supplementary file 1 [file Table_1.pdf]

Supplementary Information

CRISPR/Cas9 and Nanotechnology Pertinence in Agricultural Crop Refinement

Banavath Jayanna Naik[1], Ganesh Shimoga[2], Seong-Cheol Kim[1], Mekapogu Manjulatha[3], Chinreddy Subramanyam Reddy[4], Ramasubba Reddy Palem[5], Manu Kumar[6], Sang-Youn Kim[2]*, and Soo-Hong Lee[5]*

[1]Research Institute of Climate Change and Agriculture, NIHHS, Rural Development Administration, Jeju 63240, Republic of Korea

[2]Interaction Laboratory, Future Convergence Engineering, Advanced Technology Research Center, Korea University of Technology and Education, Cheonan-si 31253, Chungcheongnam-do, Korea

[3]Floriculture Research Division, Rural Development Administration, Wanju-gun, Jeollabuk-do, 55365, Republic of Korea

[4]CSSR and SRRM degree and PG College, Kamalapuram, Kadapa-516003, India

[5]Department of Medical Biotechnology, Dongguk University Biomedical, Campus 32, Gyeonggi 10326, Republic of Korea

[6]Department of Life Science, College of Life Science and Biotechnology, Dongguk University, Goyang 10326, Korea

*** Correspondence:**
Corresponding Authors
sykim@koreatech.ac.kr and soohong@dongguk.edu

Table: S1. Partial list of genes of cereal/food crops and its specific functions modified *via* CRISPR/Cas9 system.

Sl.No	Crop Name	Gene Name	Function	Reference
1	Rice	*OsACC-T1*, *OsALS-T1*, *OsCDC48- T3*, *OsDEP1-T1*, *OsDEP1-T2*, and*OsNRT1.1B-T1*	Disease resistance and abiotic stress tolerance	Li *et al.*, 2018e
2		*TIFY1b* and *TIFY1a*	Low temperature stress tolerance	Huang et al.,2017
3		*OsSWEET11*, *OsSWEET13*, and *OsSWEET14*	Resistant to both sheath blight and bacterial blight disease	Zafar et al.,2020
4		*OsBADH2*	Production of aroma	Ashok Kumar et al.,2020
5		*OsPRX2*	Stomatal closure/opening under the K^+-deficiency tolerance	Mao et al.,2018
6		OsSPL16	Improves grain yield	Usman et al.,2021
7		*GW2*, *5*, and *6*	Improves seed weight	Chen et al.,2020

8		*OsCYP97A4*, *OsDSM2*, *OsCCD4a*, *OsCCD4b*, and *OsCCD7*	Increase the carotenoid accumulation and the quality	Yang et al., 2017a
9		*ACCase*	Herbicide resistant	Li et al.,2018g
10		*SBEI &SBEIIb*	Increased amylose content	Sun et al., 2017
11		*OsNramp5*	Low Cd (Cadmium) accumulation	Tang et al.,2017
12		*OsAnn3*	Cold tolerance	Shen et al., 2017
13		*OsFAD2-1*	Lipid accumulation and biosynthesis	Abe et al., 2018
14		*G34S3*, and *Gn1a*	Improvement of grain number, size and panicle architecture	Huang et al., 2018
15		*GS9*	Regulate the grain size, shape, appearance and quality	Zhao et al., 2018
16		*Wx*	Synthesis of amylose	Yunyan et al., 2019

17		*elF4G*	Resistance to rice yellow mottle virus (RYMV) and rice tungro spherical virus (RTSV)	Macovei et al., 2018
18		*ISA1*	Affects starch synthesis and endosperm development	Chao et al., 2019b
19		*OsMATL*	Haploid plant production	Yao et al., 2018
20		*OsNAC041*	Saline tolerance	Bo et al., 2019
21		*OsOTS1*	Saline tolerance	Sadanandom et al.,2019
22		*OsRR22*	Saline tolerance	Zhang et al., 2019a
23		*OsNAC14*	Water discrepancy tolerance	Shim et al., 2018
24		*SAPK1* and *SAPK2*	Water discrepancy and saline tolerance	Lou et al., 2018
25	Wheat	*EPSPS*	Herbicide glyphosate tolerance	Arndell et al.,2019
26		*TaDREB2,3* and *TaERF3*	Drought tolerance	Kim et al., 2018

This is a provisional file, not the final typeset article

27		*TaCKX2-1*, *TaGLW7*, *TaGW2*, *and TaGW8*, , *TaCKX2-B1*, *TaCKX2-D1*,and *TaCKX2-A1*	Increased grain number	Zhang et al.,2019b
28		*TaPDS*	Carotenoid and chlorophyll biosynthesis	Howells et al.,2018
29		α-gliadin family members	Diminished gluten content	Sánchez-León et al.,2018
30		*TaEDR1*	Resistance to powdery mildew	Zhang et al., 2017c
31		*TaWaxy* and *TaMTL*	Haploid plants	Liu et al.,2020
32		*TaMS45*	Male sterility	Singh et al.,2018
33	Maize	*ARGOS8*	Water discrepancy tolerance	Shi et al., 2017
34		*MS8*	Male sterility with multiple phenotypes	Chen et al.,2018
35		*Zyp1*	Encodes a central element protein of the synaptonemal complex and involve in meiosis programme	Feng et al.,2018

36		*ZmLG1*	Reduced leaf angle due to the lack of auricle and ligule development	Wang et al.,2019e
37		Dek42	Regulation of kernel development	Zuo et al.,2019
38		*ZmHKT1*	Saline tolerance	Zhang et al,2018a
39	Soybean	*Drb2a*, and *Drb2b*	Drought and saline tolerance	Curtin et al.,2018
40		*GmLHY1a*, *GmLHY1b*, *GmLHY2a* and *GmLHY2b*	Alters the height of plant and its internode length	Cheng et al.,2019
41		*GmFT2a*	Photoperiod flowering pathway	Cai et al., 2018
42		*Seed storage proteins Glyma.20g148400, Glyma.20g14620Glyma.10g246300, Glyma.20g148200Glyma.10g037100, Glyma.03g163500Glyma.19g164900, Glyma.13g123500,* and *Glyma.19g164800*	Protein storage in soybean hairy roots.	Li et al.,2019b

This is a provisional file, not the final typeset article

43		*FAD2*-2	Production of oleic acid	Al Amin et al., 2019
44		*GmLox1*, *GmLox2*, and *GmLox3*	Lipoxygenase-free plants	Wang et al.,2020
45		*GmSPL9a,b,c*	Improves yield	Bao et al.,2019
46		*GmPPD1* and *GmPPD2*	Transcriptional regulation of cell division	Kanazashi et al.,2018
47	Cotton	*GhMYB25-like A* and *D*	Development of cotton fiber	Chao et al., 2019a
48		*Gh14-3-3d*	Against Verticillium dahliae in allotetraploid upland cotton	Zhang et al., 2018e
49	Barley	*HvHPT* and *HvHGGT*	HvHPT: Decreased grain size & weight<br>HvHGGT: Blocked tocotrienol biosynthesis	Zeng et al.,2020
50		*HvCKX1*	Encode cytokinin oxidase/dehydrogenase enzyme, which catalyzes the irreversible degradation of cytokinin	Gasparis et al.,2018
51		*HvMORC1*	Resistance against fungal pathogens	Kumar et al.,2018

52		*PDS1*	Carotenoid and chlorophyll synthesis	Raitskin et al., 2019
53		*WDV1* and *dsRED*	Resistance against dwarf virus and *Geminiviridae*	Kis et al.,2019
54		*hptII (Hygromycin phosphotransferase*)	Hygromycin resistance	Lawrenson et al., 2019
55	Sorghum	*CAD and PDS*	Biosynthesis of carotenoid and chlorophyll	Liu et al.,2019
56	Cacao	*TcNPR3*	Resistance against Phytophthora tropicalis	Fister et al., 2018
57	Cow pea	*VuSYMRK*	Symbiotic nodulation phenotype	Jie et al.,2019
58	Groundnut	*AhFAD2A* and *AhFAD2B*	Encode fatty acid desaturase	Yuan et al.,2019
59		*AhNFR1* and *AhNFR5*	Root nodules to nitrogen fixation	Shu et al., 2020
60	Foxtail Millet	*SiMTL*	Haploid induction	Cheng et al.,2021

This is a provisional file, not the final typeset article

61	*Coffea canephora*	*CcPDS*	Color phenotypes	Breitler et al.,2018
62	Oilseed Rape	*SPL3* Homologus genes (*BnSPL3*-A5/*BnSPL3*-A4/*BnSPL3*-C3/*BnSPL3*-C4/*BnSPL3*-Cnn)	Floral activator	Chao et al.,2018

Table: S2. Partial list of genes of vegetable crops and its specific functions modified *via* CRISPR/Cas9 system.

S.No	Crop Name	Gene Name	Function	Reference
1	Tomato	*Procera gene (DELLA protein) and GA1*	Dominant dwarf phenotype	Tomlinson et al.,2019
2		*SlMET1 (Methyltransferase 1)*	DNA methylation	Yang et al.,2019b
3		*PL & TBG4*	Developing fruit color and weight	Wang et al.,2019a
4		*SlNPR1*	Water discrepancy tolerance	Li et al., 2019a
5		*SlCBF1*	Cold tolerance	Li et al., 2018b
6		*SlMAPK3*	Drought tolerance	Wang et al.,2017a
7		*SlCLV3, SlWUS*,and *lc*	Branched inflorescence and fruit size	Rodríguez- Leal, et al.,2017
8		*Mlo* (Mildew resistant locus)	Resistance to powdery mildew	Nekrasov et al., 2017

This is a provisional file, not the final typeset article

9		*GABA-TP1, 2, 3*, *CAT9*, and *SSADH*	Gamma-Aminobutyric acid (GABA) synthesis.	Li et al., 2018a
10		*CP* & *REP*	Resistance to tomato yellow leaf curl virus	Tashkandi et al., 2018
11		*DCL2b*	Defensive action against tomato mosaic virus	Wang et al.,2018c
12		*SOLYC0g075770*	Susceptible for Fusarium wilt disease	Prihatna et al., 2018
13		*SlMAPK3*	Susceptible for Gray mold disease	Zhang et al., 2018b
14		*SlJAZ2*	Resistance against bacterial speck disease	Ortigosa et al., 2019
15		*BZR1*	Temperature tolerance	Yin et al., 2018
16		*MYB12*	Pink fruit phenotype	Deng et al., 2018
17		*SGR1*, *LCY-EBlc*, *LCY-B1* & *B2*	High lycopene content	Li et al.,2018d

18		*SP, CLV3, WUS and GGP1*	Associated with morphology, ascorbic acid synthesis and flower/ fruit production	Li et al., 2018h
19		*SP*. *OVATE, MULT, FAS* and *CycB*	Development of flower morphology, fruit size and lycopene synthesis	ZSogon et al., 2018.
20		*CCD8*	Resistance to *Phelipanche aegyptiaca*	Bari et al., 2019
21		*SlMlo1 (Mildew Resistant Locus 1)* and *SlPelo*	Powdery mildew resistance	Pramanik et al., 2021
22		*lncRNA1459*	Repressed fruit ripening	Li et al.,2018c
23		*CrtR-b2*	Carotenoid biosynthesis	Caterina et al., 2018
24		*ARF7*	Parthenocarpic fruits	Hu et al., 2018
25		*AP2a, NOR, FUL1, TDR4 FUL2*, and *MBP7*	Delayed fruit ripening	Wang et al.,2019c
26		*SlGRAS8, SlEIN2, SlERF.E1, SlARF2 B, SlACS2*, and *SlACS4*.	Fruit development and ripening	Hu et al.,2019

This is a provisional file, not the final typeset article

27		*ENO*	Affects floral meristem size	Yuste-Lisbona et al.,2020
28	Potato	*StALS1* and *StALS2*	Starch synthesis	Veillet et al., 2019
29		*RNP (*Ribo Nucleo Protein*)*	Granule-bound starch synthase	Andersson et al.,2018
30		*StSSR2*	Steroidal glycol-alkaloids (SGAs) biosynthesis	Zheng et al.,2021
31		*StGBSS1*	Improved starch quality	Kusano et al.,2018
32		*PDS (Phytoene desaturase)* and *coilin*	Carotenoid synthesis	Khromov et al.,2018
33		*StSBE1* and *StSBE2*	Improved starch quality	Tuncel et al., 2019
34		*St16DOX*	Generation of steroidal glycoalkaloids-free hairy roots	Nakayasu et al.,2018
35	Sweet potato	*IbGBSSI* and *IbSBEII*	Improved starch quality and quantity	Wang et al.,2019d

36	Field Masturd (*Brassica campestris*)	*Pectin-methylesterase genes- Bra003491, Bra007665,* and *Bra014410*	Methylation of pectin	Xiong et al., 2019
37		*BaPDS1* and *BaPDS2*	Albino phenotype and flowering	Sun et al., 2018a
38		*CENH3*	Production of haploid inducer, termed green fluorescent protein (GFP)-tailswap	Stajic et al.,2019
39	Cabbage (Brassica *rapa subsp. Pekinensis*)	*FRI* and *PDS*	Albino phenotype and flowering	Murovec et al., 2018
40	Kale (*Brassica oleracea*)	*BoaCRTISO(Carotenoid isomerase*)	Carotenoid biosynthesis	Sun et al.,2020a
41		*BoPDS, (Phytoene desaturase) BoSRK, (S-receptor kinase)* and *BoMS1 (Male-sterility)*	Albino phenotypes, Male sterility, self-incompatibility	Ma et al.,2019a,b
42	Carrot	*DcF3H*	Anthocyanin metabolism	Chodacka et al., 2018

This is a provisional file, not the final typeset article

43		*DcPDS and DcMYB113*	Purple depigmented carrot plants	Xu et al.,2019
44	Ground cherry	*SP*, *SP5G*, and *CLV1*	Development of flower morphology and fruit size	Lemmon et al., 2018
45	Brinjal	*SmelPPO4*, *SmelPPO5*, and *SmelPPO6*	Decreased polyphenol oxidases (PPO) activity and browning of the berry flesh after cutting	Maioli et al.,2020
46	Onion	*FUM1*	Reduced fumonisin biosynthesis	Ferrara et al.,2019
47	Lettuce	*LsNCED4*	Inhibition of seed germination rate in higher soil temperature	Bertier et al.,2018
48		*CsCRUC*	Improving seed proteome and fatty acid profile	Lyzenga et al., 2019
49	Chilli	*NAC72*	Drought tolerance	Joshi et al.,2019
50	Cassava	*5-Enolpyruvylshikimate-3-phosphate synthase*	Weed tolerance	Hummel et al., 2018
